# Female Genital Mutilation in Infants and Young Girls: Report of Sixty Cases Observed at the General Hospital of Abobo (Abidjan, Cote D'Ivoire, West Africa)

**DOI:** 10.1155/2014/837471

**Published:** 2014-03-04

**Authors:** Kouie Plo, Kouadio Asse, Dohagneron Seï, John Yenan

**Affiliations:** ^1^Department of Pediatrics, General Hospital of Abobo, 14 PB 125 Abidjan 14, Cote D'Ivoire; ^2^Department of Pediatrics, University Teaching Hospital of Bouake, 01 BP 1174 Bouake 01, Cote D'Ivoire

## Abstract

The practice of female genital mutilations continues to be recurrent in African communities despite the campaigns, fights, and laws to ban it. A survey was carried out in infants and young girls at the General Hospital of Abobo in Cote D'Ivoire. The purpose of the study was to describe the epidemiological aspects and clinical findings related to FGM in young patients. Four hundred nine (409) females aged from 1 to 12 years and their mothers entered the study after their consent. The results were that 60/409 patients (15%) were cut. The majority of the young females came from Muslim families (97%); the earlier age at FGM procedure in patients is less than 5 years: 87%. Amongst 409 mothers, 250 women underwent FGM which had other daughters cut. Women were mainly involved in the FGM and their motivations were virginity, chastity, body cleanliness, and fear of clitoris similar to penis. Only WHO types I and II were met. If there were no incidental events occurred at the time of the procedure, the obstetrical future of these young females would be compromised. With FGM being a harmful practice, health professionals and NGOs must unite their efforts in people education to abandon the procedure.

## 1. Introduction 

Female genital mutilation or clitoris cutting (FGM) is defined as the partial or total removal of clitoris and labia. Well known since antiquity in Egypt, this practice is widespread in the world but mainly in Africa [1–7]. Many factors related to tradition, sexual behavior in the males, and religious beliefs impact on FGM [8–11]. The clinical observation of three cases: first in female newborn twins aged three weeks and second in an 8-year girl led us to carry out a prospective study on FGM in infants and young girls. We studied the prevalence and etiologic factors in the pediatric ward at the General Hospital of Abobo, a suburb of Abidjan. Our study also aimed to influence the parents of girls and the traditional circumcisers and practitioners to abandon the practice. Not only are there laws prohibiting FGM, but there are later gynecological and obstetrical consequences of FGM.

Our objectives were (1) to identify FGM in infants and young girls seen in our clinic, (2) to describe the sociocultural context of young girls who had undergone FGM, (3) to assess the mothers' FGM status and attitudes regarding the practice, (4) and to determine the clinical issues in terms of immediate or later complications.

## 2. Patients and Methods

The General Hospital of Abobo is the premier public health entity that takes care of many patients of Abobo and others from periurban areas of Abidjan. This includes about 1,500,000 inhabitants or 20% of the population of the city of Abidjan. The pediatric ward enrolls 8,000 outpatients and hospitalizes about 2,500 inpatients a year. Eligible for the survey were infants and young girls seen at the hospital for any reason and whose mothers agreed verbally or by written consent to enter the survey and answer the questionnaire items.

From 16 April to 16 December, 2007, during eight months, 409 infants and young girls aged from 1 to 14 years and their mothers entered the prospective study. The FGM status of girls was recorded during in- or outpatient visits. These examinations were complemented by a questionnaire comprising four groups of items: (1) patient's identification with age, native region (north, south, east, west, and center of the country), ethnic group, religion, and nationality; (2) history and circumstances of FGM practice, age at FGM procedure, observance of ritual ceremony or not, the individuals behind the decision of FGM practice, and the motivations; the circumciser (childbirth attendant or matron), tools used for FGM, and the occurrence of immediate complications such as bleeding, the medicines used to heal the wounds, the rituals observed during the FGM ceremony, time elapsing before full wound healing, and the FGM status of the mothers themselves; (3) evidence of FGM by a physical general examination including the genitalia and classification of FGM, when present, according to the World Health Organization's (WHO) classification [12]: these are type I: Sunna partial or total removal of the clitoris and/or the prepuce or excision, type II: partial or total removal of the clitoris and the labia minora, with or without excision of the labia majora (clitoredectomy), type III: narrowing of the vaginal orifice with creation of a covering seal by cutting and apposing the labia minora and/or the labia majora with or without excision of the clitoris (infibulations or pharaonic circumcision), and type IV: all other harmful procedures to the female genitalia for nonmedical purposes piercing, pricking, incising, scraping, and cauterization of the genitalia area (unclassifiable).

*Ethics of Our Study.* The General Hospital Consultative Committee that evaluates the relevance, feasibility, confidentiality of the information obtained, and ethnical aspects of clinical research reviewed our protocol of research and gave its permission. Once the mother's oral or written consent was obtained, the same female physician organized the questionnaire, performed the physical examinations, and filled out the data sheets during both inpatients and outpatients consultations.

## 3. Results

Sixty of 409 infants and young girls (15%) were diagnosed as having undergone FGM. Their age distribution at the time of consultation or hospitalization was between 1 and 5 years: 19, (32%) between 5 and 10 years: 29 (48%) and between 10 and 15 years: 12 (20%). The baseline characteristics on epidemiological aspects were the earlier age at FGM practice, 19 infants under one-year old; women were the individuals behind the decision of FGM practice (96.60%); several West African ethnic groups from Cote d'Ivoire, Mali, Burkina Faso, Benin, and Niger were implicated; Muslim families 59/60 and the illiterate or low educational level of the parents 81% and 87%, respectively in mothers and fathers were found as major factors. The practitioners were traditional circumcisers; no nurses, midwives, or physicians were involved.

**Figure 1:**
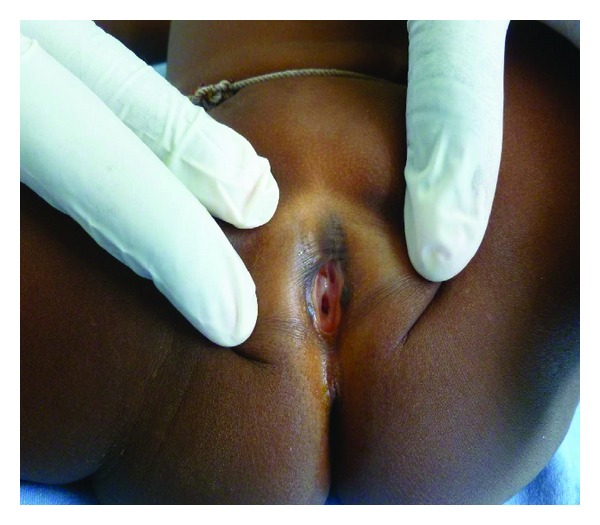
FCM type II in a 1-year-old Peulh female (picture photographed in our ward of pediatrics).

About the clinical findings, only FGM types I and II were diagnosed. Pain, fever, and minimal bleeding were the main symptoms and signs disclosed by the mothers surveyed. There was a potential relative risk of undergoing type II mutilation for those under five years of age. Amongst the mothers, 250 women out of 409 had had FGM (61.1%). Among them, 151 had their daughters (60.4%) undergone the procedure. The details of sociocultural characteristics of our samples and the clinical findings of our patients are reported on the Tables 1, 2, and 3 and Figure 1 shows a FGM type II in a 1-year-old Peulh female.

## 4. Discussion 

### 4.1. Epidemiology

The prevalence of FGM among our patients population was 15%. The main associated factors were as follows: women were the decision makers relative to FGM; in 97% of cases, it was a grandmother, mother, or aunt who initiated the operation. Their chief motivations encompassed chastity (100%) and esthetics (68%).

FGM was encountered most often in the communities of three countries with a relative high rate in some ethnic groups as the Malinke and Senoufo. Most families were Muslim (98.3%) and most parents were illiterate, 81% and 87% in mothers and fathers, respectively.

Previous reports on FGM in West African women [13–17] gave rates varying from 45 to 60% in the general population and 20 up to 87% in northern and western regions of the country. Rates were high among the Dan, Malinke, Wè, and Senoufo ethnic groups. The proportion of young girls has been estimated by Oulaï in his report to be about 500 females. Those were 31% for 155 children between 0 and 5 years; 31.4% for 157 between 12 and 16 years of age; and 38.6% in 193 women.

The majority were of the Dan group (80.12%); 94.6% were illiterate. Only 5.4% had had a primary and secondary school level of education. The religions were distributed in this way: Christians: 55.91%, Muslims: 43.34%, and animists 0.75% [18]. The decision makers were mainly women: grandmothers (71.6%), mothers (25.0%), and fathers (3.4%). In a survey carried out in 38,816 Egyptian young school girls the prevalence of FGM was 50.3% (19,543). The motivations were religion: 33.4%, cleanliness for girls: 18.9%, cultural and ancient tradition: 17.9%, and chastity 15%. Compared to the study of Snow et al. in Nigeria, similar characteristics had been found amongst young girls and women between 15 and 49 years victims of FGM and interviewed: age at FGM prior one year 371 (68.3%), between one and ten years 43 (7.9%), and ten to twenty years 88 (16.3%) [19]. The religious context in this study was Pentecostals: 562 (33.1%), Protestants 277 (16.3%), Catholics 613 (36.1%), Muslim 100 (5.9%), and others 146 (8.6%). On the other hand educational level of the surveyed girls was distributed as follows: primary: 330 (19.3%), secondary: 533 (32.2%), tertiary 767 (44.9%), and none 77 (4.5%) showing the inhomogeneous and spatial distribution of sociocultural factors in the practice of FGM. The commonest basis would be the ancient and tradition beliefs [20, 21]. These observations are similar to those in data from Burkina Faso, Mali, Guinea, and Gambia in West Africa [22].

### 4.2. Clinical Findings and FGM Classification

About the clinical findings, FGM types I and II accounted for 100% of cases whereas in Somalia, Sudan and Egypt, Mali, and Burkina Faso types III and IV were mentioned up to 89% [23–25]. The immediate complications, such as pain, fever, and minimal or incidental bleeding as short period of bleeding (if it is severe) could be catastrophic, were probably underestimated. Hemorrhages, infections, and death have been reported together with the posttraumatic stress disorders and memory problems [26, 27]. What is the future of our patients? Most did not have major long-term complications, after the fear and the psychological trauma of FGM, finding similar to those of Althaus [28].

These infants and young girls, once adults, could nonetheless face the late consequences of FGM. These include psychological, gynecological, and obstetrical difficulties. Painful intercourse, bleeding, dystocia, long labor, and episiotomy needed at the time of labor have been reported by gynecologists and many nongovernmental organizations fighting for the abandonment of FGM [29, 30].

Although in our study no major psychological troubles were encountered, posttraumatic stress disorders have been reported in patients similar to ours [31–35].

### 4.3. Elimination of FGM

Many African countries and elsewhere in the world have laws prohibiting FGM. Nongovernmental organizations (NGOs) campaigns against FGM continue to fight for its abandonment [36–40]. Despite these laws and campaigns to eliminate it, FGM continues in urban and rural areas, as our study and other recent reports have shown [41, 42].

## 5. Conclusion

Our study has shown the current reality of FGM in earlier age. It resulted from traditional and religious beliefs. Women, having a past history of FGM, play the key role in the occurrence of FGM in their daughters. Only types I and II mutilations have been met.

There were few immediate complications. The combination of law-enforcement together with information and education activities by NGO's aimed at female populations has curtailed FGM in most countries. Continuing efforts are needed to eliminate FGM as a threat to the health and wellbeing of women.

## Figures and Tables

**Table 1:** Main characteristics of patients' parents.

Parents' characteristics	Number (%)	
Nationality		
Cote d'Ivoire	35 (58.3)	
Mali	18 (30.0)	
Burkina Faso	5 (8.3)	
Benin and Niger	2 (3.4)	
Ethnic groups		
Malinke and Senoufo (Cote d'Ivoire)	35 (58.3)	
Malinke, Bambara, Dogon, and Peulh (Mali)	18 (30.0)	
Senoufo (Burkina Faso)	5 (8.3)	
Hausas (Benin and Niger)	2 (3.4)	
Religion		
Muslim	59 (98.3)	
Christian	1 (1.7)	
Level of education	Mothers	Fathers
Analphabets	40 (66.7)	33 (55.0)
Primary school	9 (15.0)	19 (31.7)
Secondary school	6 (10.0)	5 (8.3)
High school	5 (8.3)	3 (5.0)
Decision makers		
Grandmothers	43 (71.6)	
Mothers and aunts	15 (25.0)	
Fathers	2 (3.4)	
Parents' motivations		
Virginity and chastity	60 (100.0)	
Body cleanliness	38 (63.3)	
Clitoris: male organ and harmful	38 (63.3)	

**Table 2:** Characteristics of infants and young girls undergone FGM.

Patients' characteristics	Number (%)
Age at FGM practice (years)^1^	
<1	19 (31.7)
(1–5)	29 (48.3)
(5–10)	12 (20.0)
FGM classification (WHO)	
Type I	8 (13.3)
Type II	52 (86.7)
Symptoms reported after FGM^2^	
Pain	60 (100.0)
Fever	47 (78.3)
Minimal bleeding	60 (100.0)

^1^Areas where FGM took place: Abobo and Adjamé (35 or 58% in Abidjan); Korhogo, Ferkessedougou, Odienne, and Boundiali: 11 (Northern region of Cote d'Ivoire); Kaye, Mopti, Boroni, and Bonangoro 14 (23%) in Mali. All the FGMs were performed in circumcisers' home with knives, razors, or blades.

^2^Each girl could have one or more symptoms.

**Table 3:** Distribution of patients according to the age at FGM practice and FGM types.

Age at FGM practice (years)	FGM classification (WHO)		
	Type I	Type II	Total
<1	0	19	19
1–5	1	28	29
5–10	7	5	12

Total	8	52	60

There is a high risk to have undergone FGM type II between 1–5 years (IC 95% *P* value < 0.05).
